# Selecting the most informative positive and negative controls for self-controlled case series (SCCS): Rationale, approach, and lessons from studies investigating the safety of COVID-19 vaccines

**DOI:** 10.7189/jogh.14.03037

**Published:** 2024-08-06

**Authors:** Igor Rudan, Srinivasa Vittal Katikireddi, Steven Kerr, Tristan Millington, Zoe Grange, Christopher Sullivan, Adeniyi Francis Fagbamigbe, Ben Swallow, Amanj Kurdi, Kirsty Morrison, Karen Jeffrey, Colin R Simpson, Lewis Ritchie, Chris Robertson, Aziz Sheikh

**Affiliations:** 1 Usher Institute, The University of Edinburgh, Edinburgh, UK; 2 Algebra University College, Zagreb, Croatia; 3 MRC/CSO Social & Public Health Sciences Unit, University of Glasgow, Glasgow, UK; 4 Public Health Scotland, Glasgow, UK; 5 School of Medicine, Medical Sciences & Nutrition, University of Aberdeen, Aberdeen, UK; 6 School of Mathematics and Statistics, University of St Andrews, St. Andrews, UK; 7 Strathclyde Institute of Pharmacy and Biomedical Sciences, University of Strathclyde, Glasgow, United Kingdom; 8 Department of Clinical Pharmacy, College of Pharmacy, Hawler Medical University, Erbil, Iraq; 9 College of Pharmacy, Al-Kitab University, Kirkuk, Iraq; 10 School of Pharmacy, Sefako Makgatho Health Sciences University, Pretoria, South Africa; 11 School of Health, Wellington Faculty of Health, Victoria University of Wellington, Wellington, New Zealand; 12 Department of Mathematics and Statistics, University of Strathclyde, Glasgow, UK; 13 Nuffield Department of Primary Care Health Sciences, University of Oxford, Oxford, UK

The self-controlled case series (SCCS) is a study design used in epidemiology to test for variation in outcomes for a group of individuals before and after an exposure or intervention, with each pre-intervention individual serving as a control for themselves in the post-intervention period. The design is particularly useful for studying rare events or outcomes that occur in a time-dependent manner, such as uncommon side-effects of a medical intervention [1–3]. In the Early Pandemic Evaluation and Enhanced Surveillance of COVID-19 (EAVE II) study, we used this SCCS design to evaluate the safety of coronavirus disease 2019 (COVID-19) vaccines in various age groups of the Scottish and UK populations [4–13].

The SCCS design involves using data only from individuals who have the outcome of interest; for this reason, the study population for analysis consists of individuals who have experienced the outcome and, in most cases, the exposure or intervention of interest. However, it is also possible to include individuals with the outcome, but not the exposure of interest, which can further strengthen the study design if the additional information obtained in this way helps answer the research question [1–3]. Controls who are either exposed or not exposed will not contribute to the precision of comparisons between the periods before and after exposure in those who experienced the outcome. However, they can still serve to increase the precision of the different estimates in the period where there was or was not an exposure.

Each study subject can thus serve as their own control; however, this typically requires the effect of the exposure to be transient. In SCCS, this approach eliminates the need for external unexposed control groups. The design takes advantage of within-person comparisons over time. It compares the occurrence of the outcome (incidence rate) during exposed (risk) periods to the occurrence of the outcome (incidence rate) during baseline (control, unexposed) periods within the same individuals. In vaccine safety studies, the exposure (risk) period is a short period of time following vaccination; the baseline (control, unexposed) period is usually a period of time before vaccination, but may also include time after the exposure period has ended, as the effect of the exposure is assumed to be transient. This allows for the calculation of the incidence rate ratio (IRR) [1–3]. We recap these standard features of SCCS before discussing a beneficial addition to the typical SCCS methodology: incorporating positive and negative controls.

Positive controls, where there is known to be a causal relationship between the exposure and outcome [14], and negative controls, where there is no such causal relationship between the exposure and outcome [15], are control comparisons that can be used to help detect the presence of residual confounding. It is important to note that there can be different types of positive or negative controls: these can be exposure-based or outcome-based. In this regard, they play a similar role to positive or negative controls in laboratory assays [16,17].

## KEY ELEMENTS AND LIMITATIONS OF SCCS

The typical SCCS design includes five steps. First, researchers define an observation period over which cases and events are sampled. They then select cases by identifying individuals who have experienced the outcome of interest within a defined population, such as patients with a specific disease or condition or individuals residing within a defined geographical area. After this, they select exposure (risk) periods by identifying specific time periods during which the exposure of interest occurred for each study subject. This selection is usually based on medical records or other relevant data sources. Following this step, they select baseline (control, unexposed) periods; these are chosen within the same individuals, representing periods when the individual was not exposed to the intervention or had a different exposure status. These control periods serve as the baseline for comparison. Usually, these periods are just observation time not included in exposure periods. In this, the baseline period is often a subset of the unexposed time: researchers might not choose the whole of the unexposed time as a control period because having the event can affect whether the person is subsequently exposed, which could lead to the event rate in the baseline period being biased. As an example in the context of vaccine safety, it is common to exclude the period 14 days before vaccination because the recipients need to be healthy in this period to get the vaccine. The definition of both the exposure (risk) period and baseline (control, unexposed) period depend on pre-existing knowledge of both biological mechanisms and clinical manifestations of the exposure and outcome. It is important to choose an exposure (risk) period that allows for adequate latency between exposure and outcome. Lastly, during analysis, researchers compare the occurrence of the outcome during the baseline (control, unexposed) periods is compared to its occurrence during exposed (risk) periods within each study subject. In doing so, they use statistical methods such as conditional logistic or Poisson regression to estimate the association between exposure and outcome [1–3,14–17].

The SCCS design is advantageous because it controls for time-invariant unmeasured confounding factors that may arise from differences between individuals given that in SCCS each individual serves as their own control. Age variation in adults is not usually as important when observation periods are brief, and any potential effect modification can be investigated by fitting interactions. Therefore, using short time periods is important to avoid confounding by age. Clearly, the SCCS design is particularly useful for studying outcomes that have short latency periods to limit the possible effect of unmeasured confounders, or that are rare enough that traditional cohort or case-control studies may be impractical, too expensive. or entirely unfeasible [1–3]. However, in vaccine studies in children, age may be an important time-varying confounder that needs to be allowed for in the SCCS model [4]. Another possible issue is if the outcome is linked to high mortality rates, which can lead to various survival biases [1].

Therefore, SCCS studies do have certain limitations. A key one is the assumption that there would be no unmeasured time-varying confounders which overlap with exposure (risk) and baseline (control, unexposed) periods in the SCCS. However, the presence of such confounders may introduce substantial bias. For this reason, SCCS studies are often most appropriate when the latency period between exposure and outcome is short. Also, time-varying confounders need to be adjusted for, but that applies to most study designs in epidemiology. Issues mainly arise when it is impossible to adjust because there are insufficient data.

It is important to note that, fundamentally, the target population for inference in SCCS is the entire population, including both those affected and those who do not have the outcome. Conditioning on the occurrence of an event of interest eliminates all constant influences for an individual during the entire period of study. Consequently, only the affected cases are required to estimate the parameters of interest. However, the full model could also work without conditioning by using all the information from both affected and unaffected; if we could perfectly adjust for all variables that are constant in time, we would expect to get the same estimates as the model that conditions on having an event of interest.

So, although the SCCS design estimates the modelled rate ratio in the entire population, including both cases and non-cases, one should always consider whether SCCS is generalisable to the broader population. Namely, SCCS study designs are often dependent on highly specific populations that have the outcome of interest and may not represent the general population perfectly. However, this concern might be more theoretical than practical, because – as we explained earlier – selecting cases for analysis does not induce bias and does not inherently impact generalisability. Indeed, the SCCS is derived mathematically from a cohort model that includes strata terms for each individual, so it should yield the same inferences as such model [1].

Notwithstanding the potential limitations, the SCSS is now widely regarded as a valuable study design for investigating associations between exposures or interventions and outcomes within individuals over time [1–3,16,17].

## RATIONALE FOR AND APPROACH TO THE USE OF POSITIVE CONTROLS IN SCCS

If there is known to be a causal relationship between an exposure/intervention and an outcome (e.g. certain safety issues for a vaccine), then positive controls can be included in a SCCS study. Positive controls would be those with known and scientifically proven outcomes that are expected to be associated with the exposure/intervention. In the case of EAVE II studies, the exposure of interest was COVID-19 vaccinations. Positive controls can be included to demonstrate that the study design and analytical methods can detect known associations, thus helping to validate the study's findings. They provide a benchmark against which the observed associations in the study can be compared, helping to distinguish between true vaccine-related events and coincidental occurrences in vaccine safety studies.

To choose suitable positive controls for SCCS, a review of the scientific literature would be recommended to identify well-documented and preferably scientifically explained side effects or events associated with the vaccines under study. Guidance from health regulatory agencies like the Centers for Disease Control (CDC) and the World Health Organization (WHO) recording known vaccine-associated events may prove useful in this regard, as can data on known side-effects documented by vaccine manufacturers. Live attenuated vaccines might have different known events compared to inactivated vaccines and mRNA-based vaccines, so vaccine type can also be important [18].

In our EAVE II studies, the chosen positive controls also had to be relevant to particular age groups, because it is known that certain vaccine adverse reactions might be more common or exclusively present in certain age. We needed to choose events with a clear and defined temporal pattern post-vaccination and include a mix of both common and rare, but serious events, if these were identified in time. That allowed us to explore adverse events across a spectrum of frequencies and severities.

Once positive controls are selected, their analysis needs to be integrated into the SCCS-based study to validate and strengthen the results of vaccine safety study. The goal of adding positive controls is to demonstrate that if there were a vaccine-related safety issue such as, for example, vaccine-induced myocarditis, the study design and analysis could detect it.

Examples of studies where positive controls were used include investigations of neurological complications after first dose of COVID-19 vaccines and SARS-CoV-2 infection [7]; studies exploring the risk of thrombocytopenic, haemorrhagic, and thromboembolic disorders following COVID-19 vaccination and positive test in Wales [11]; and studies on safety outcomes following COVID-19 vaccination and infection in 5.1 million children in England [19]. In these studies, the risk of vaccine-induced anaphylaxis, known to be associated with vaccination, was assessed by extracting any clinical record for anaphylaxis. Anaphylaxis showed the expected increased risk in the 0–7 days after the first dose for both vaccines, but the elevated risk did not persist beyond that time period, which is consistent with biological understanding [7].

## RATIONALE FOR AND APPROACH TO THE USE OF NEGATIVE CONTROLS IN SCCS

Negative control (or ‘falsification’) variables are used to detect residual confounding by seeking evidence of associations between variables that are believed to be closely linked to confounders, but not to either the exposure/intervention or the outcome [20,21].

Negative controls in SCCS should be causally related to confounders of concern and to either the exposure or the outcome – but not both. Here, the terminology ‘negative control’ is inherited from laboratory experiments such as assays, where a set of negative controls is run in parallel to validate the test. The reasoning behind introducing negative controls is similar to the rationale for specificity of an exposure-outcome relationship being seen when assessing causality within the Bradford Hill framework [16].

Negative control outcomes are therefore variables which should not be caused by the exposure or intervention under investigation, but are affected by potential confounders, e.g. studying suicide as an outcome of smoking [22]. Similarly, negative control exposures are variables which are affected by relevant confounders, but should not plausibly affect outcomes. An example is studying the effects of time periods before, rather than after, a national policy starts, i.e. comparing the outcome rates in two time periods when exposure was not present [3,17].

While the use of negative control variables has increased, they remain under-utilised and there is arguably potential to increase their usage within SCCS designs [17,23]. For example, when using an SCCS design to study vaccine safety, there are a few potential approaches for incorporating appropriate negative control groups [4,7,11].

For studying vaccine safety, unexposed periods, i.e. prior to receiving vaccine, will be the ‘control’ periods within the same study subjects. These unexposed periods would represent time intervals when the subject has not received the vaccine, the safety of which is being investigated. Comparing the occurrence of hospitalisation during exposed periods (after vaccination) to unexposed periods (prior to vaccination) will enable assessment of the safety of the vaccine, while comparing the rates in the unexposed periods will provide evidence on potential temporal confounding.

An example of negative control exposure is as follows: if a COVID-19 vaccination programme was delivered at around the same time as a flu outbreak, then the SCCS could be confounded by flu infections, which is a time-varying confounder. The negative control exposure would therefore use the date of influenza infection, rather than the date of vaccination, as the beginning of the exposure period. In more complex SCCS analyses, it is even possible to have the negative control and the exposure in the same analysis.

Similarly, if studying severe COVID-19 outcomes in the context of a pandemic, there may be a possibility that changes in bed availability might influence propensity to be hospitalised over time. Researchers might try to capture this effect by including a temporal effect in the model, but whether this is adequate might be unclear. So, they could look at cause-specific hospitalisations for outcomes that should not be affected by the COVID-19 vaccine, e.g. hospitalisations for other infectious agents.

Similarly for vaccine safety, hospitalisation for a serious side effect is the outcome in a typical SCCS. Here, too, adding a group who were also hospitalised, but for an entirely unrelated reason, may enhance the interpretability of the results. To check for potential confounding or bias in our studies, we considered events related to hip fractures as negative control outcomes in older adults [11]. We also examined the associations of exposures with coeliac disease as a negative control outcome and found no increased risk of coeliac disease across the pre-specified time periods for the vaccine exposures, but a decreased risk on the day of vaccination [7]; and we studied hospitalisations for poisoning in children [4]. Clinically, these events are unlikely to be directly caused by vaccination.

If there were differences in thresholds for hospitalisation over time (e.g. due to changes in bed availability), this could result in greater risk of hospitalisation for both the outcomes of interest and the negative control outcomes. It is possible that admissions for traffic accidents or sports injuries would also be acceptable and useful alternative negative control outcomes.

For all analyses, the choice of negative control group will depend on the specific research question, feasibility, and availability of data, and each approach will have its own advantages and limitations [22–24].

## FURTHER CONSIDERATIONS

By design, an SCCS addresses time invariant confounding, so residual confounding will potentially arise from effects that are time varying. Including unexposed individuals in SCCS studies can improve estimation of the age or period effect. However, they may need to be followed up in at least two different types of periods to contribute any information to the analysis. Vaccine safety can be additionally assessed by, for example, a SCCS of population groups where there may be less data and therefore more noise, such as children and young people in COVID-19 [4], by supplementing self-controls with unexposed cases to reduce noise in the baseline (control, unexposed) group. This could help with estimating the associations between the intervention and the outcome more precisely, assuming the unexposed cases have a similar demographic composition to the self-controls [22–24].

Also, another complicated issue is the potential for collider bias arising from the sampling. Collider bias can result from the situation when an exposure and an outcome independently cause a third variable – a ‘collider.’ Inappropriate controlling for a collider variable can occur in study design or statistical analysis, inducing a distorted association between the exposure and outcome, when in fact none exists. Collider bias occurs mainly in observational studies and can be induced by sampling [25].

In the context of studying vaccines, although SCCS study designs are predominantly used to evaluate their safety, they can also be used to study their effectiveness, as shown by published examples [26,27].

## FURTHER EXAMPLES

In our EAVE II research, we linked Scottish COVID-19 vaccination, general practice consultation and mortality data, as well as the information on hospital admissions for poisoning in the age group of interest by using unique identifiers. In one such study, we used an SCCS design to evaluate the safety of the BNT162b2 vaccine among 12–17-year-olds in Scotland [4] based on the national data on hospital admissions and general practice consultations. The analysis included all vaccinated 12–17-year-olds in Scotland, with 29 potential adverse events of special interest (AESI) chosen for safety analysis.

This example shows how the time periods of interest are important in SCCS design and need to be carefully defined. We calculated the number of hospital stays for the AESI and for poisoning that occurred in a baseline period (75 to 15 days before the first dose BNT162b2 vaccination and during defined risk periods following vaccination) for every individual, for each vaccine dose number, and for each health condition. As a result, we used the SCCS design to study the temporal association between the first and second dose BNT162b2 and 17 AESI health outcomes in 12–17-year-olds in Scotland.

We included all hospital stays in the periods to study both incident cases and exacerbations of existing conditions. For the statistical analysis, we fitted conditional Poisson models while considering hospital stays stratified by individual, with an offset equal to the logarithm of the length of the period. We further estimated IRRs to quantify the rate of hospital stays for a health outcome in the risk period following vaccination relative to the baseline period (75 to 15 days before first dose BNT162b2 vaccination), with an IRR >1 suggesting an increased rate of hospitalisation following vaccination. Individuals were censored on the earliest of the following: date of death, study end date (30 April 2022) or (with respect to their first dose) date of second dose BNT162b2 vaccine. We only conducted the SCCS analysis for outcomes where at least five hospital stays were recorded in the risk period following vaccination for a given health outcome [4].

These methods were used in an additional analysis of general practice consultations for myocarditis and pericarditis as the health outcome, as these were the most frequently reported outcomes of concern in previous studies on vaccine safety [4]. We found no significant increase in the rate of hospital stays following first or second dose vaccination, nor was there a detectable increase of myocarditis and pericarditis in the general practice data. However, the observed numbers were very small and larger studies are required to confirm our initial observations.

Unvaccinated 12–17-year-olds who experienced the health outcome with admission date during the period 1 September 2020 to 30 April 2022 were also included in the SCCS analysis, with an additional temporal stratification of calendar time to allow for any potential trends in the health outcomes over time. This, in effect, was an adjustment for temporal confounding detected by the use of time periods as negative exposure controls in the unexposed period. To add negative outcome controls to the SCCS design, we examined the associations of exposures with hospital admissions for poisoning in children and young people as a negative control outcome. We found no increased risk of poisoning in the 1–90 days following vaccine exposure, which strengthened evidence for the conclusion of the primary analysis [4].

## CONCLUSIONS

Positive and negative controls are helpful additions to the traditional SCCS design, as they can help inform assessments of the potential bias related to lack of adjustment for unmeasured time-varying confounders. They can also expose other issues, such as selection biases, which may be a feature of the data sets. These ‘controls’ are not individual cases, but rather control comparisons – different exposure-outcome pairs in which the association is known. Thereby, the terms ‘positive’ and ‘negative’ control relate to the presence or absence of an association, and not to whether the exposure or outcome differ from the primary analysis (personal correspondence with Paddy Farrington, 15 March 2024).

For the control comparison to be useful, it needs to reflect the analysis of interest in some relevant way. Often, in order to keep as close as possible to the question of interest, the positive/negative controls will share either the exposure or the outcome, with the exposure/outcome pair that the researchers are actually interested in. However, this does not need to be the case in every study – the control comparison just needs to be relevant, so that any biases present in the main study are likely to also be present in the analysis of controls (personal correspondence with Paddy Farrington, 15 March 2024).

It is always advisable to carefully consider the study design and analysis plan of SCCS to minimise potential confounding effects. By adding positive and negative controls to a typical SCCS study design, researchers can enhance its rigour, detect potential bias due to residual confounding, and improve the validity of the findings. However, SCCS studies also have their limitations – they are often difficult to set up, involve additional data collection/analysis, can be underpowered, and do not necessarily eliminate the potential for residual confounding.
